# Synthesis, characterization, and physico-chemical aspects of a new PVC-based quaternary triethanol ammonium chloride anionite for tungsten recovery

**DOI:** 10.55730/1300-0527.3678

**Published:** 2024-03-13

**Authors:** Bahig ATIA

**Affiliations:** Department of Geology of Isotopes, Research Sector, Nuclear Materials Authority, El Maadi, Cairo, Egypt

**Keywords:** Tungsten recovery, triethanol amine (TEA), PVC-TEAC anionite

## Abstract

The usability of polyvinyl chloride-based quaternary triethanol ammonium chloride anionite (PVC-TEAC) as a potential extractant for tungstate was investigated to recover tungstate from Gabal Qash Amir, Egypt, assaying 70.91% WO_3_. Structure elucidation for PVC-TEAC anionite was successfully carried out using several techniques. Experimental measurements, such as pH, agitation time, initial tungsten concentration, anionite dose, co-ions, temperature, and eluting agents, have been optimized. It was found that PVC-TEAC anionite has a maximum capacity of 63 mg per gram. From the distribution isotherm modeling, Langmuir’s model fits the experimental results better than Freundlich’s, with a theoretical value of 61.728 mg g^−1^. According to kinetic modeling, the first- and second-order modeling may be regarded as a mixed modeling for a successful adsorption system. Thermodynamic prospects reveal that the adsorption process was predicted as an exothermic, spontaneous, and preferable adsorption at low temperatures. Tungsten ions can be eluted from the loaded anionite, by 1M H_2_SO_4_ with a 97% efficiency rate. It was found that PVC-TEAC anionite reveals good separation factor (S.F.) towards most of co-ions. A successful Alkali fusion with NaOH flux followed by tungstate recovery by PVC-TEAC anionite is used to obtain a high-purity tungsten oxide concentrate (WO_3_), with a tungsten content of 78.3% and a purity of 98.75%.

## 1. Introduction

Tungsten, also known as wolfram, is an element with the highest melting point, reaching an impressive 3422 °C. With the symbol W and atomic number 74, tungsten stands out as a remarkable metal in the periodic table. Its density of 19.25 g cm^−3^ makes it one of the heaviest metals known to man. Not only does tungsten possess exceptional physical properties, but it also exhibits impressive thermal and electrical conductivity. Compared to other metals, tungsten has a low vapor pressure, which contributes to its stability and durability. Additionally, it boasts high moduli of compression and elasticity, further enhancing its strength and resilience [1]. Tungsten is a vital metal in thermo-emission applications, owing to its exceptional electron emissivity. This property is attributed to the presence of trace elements in tungsten. Additionally, tungsten is highly stable, both chemically and thermally, making it an ideal choice for various industrial processes. Despite its numerous advantages, tungsten usually contains small amounts of carbon and oxygen, which can make it brittle and hard. This can pose challenges in certain applications, but with proper handling and processing, these issues can be mitigated [2]. The average abundance of tungsten in the Earth’s crust is approximately 1.5 parts per million (ppm), making it much rarer than most rare earth elements (REEs). Naturally occurring primary tungsten minerals can be categorized into two groups: the wolframite group and the scheelite group. The wolframite group consists of minerals such as wolframite [(Fe,Mn)WO_4_], hübnerite (MnWO_4_), ferberite (FeWO_4_), and sanmartinite [(Zn,Fe)WO_4_] [3]. The scheelite group includes minerals like scheelite (CaWO_4_), stolzite, and raspite (PbWO_4_). However, it is important to note that only wolframite and scheelite are abundantly available and economically significant. The remaining tungsten minerals are rare and typically found in trace amounts [4]. Secondary tungsten minerals are typically believed to be formed through hydrothermal or supergene alteration of primary tungsten minerals rather than through atmospheric weathering [5]. The effects of hydrothermal alteration on primary tungstate minerals, such as ferberite and scheelite, can give rise to various secondary tungsten minerals. These include ferritungstite [(W,Fe)(O,OH)_3_], aluminotungstite [(W,Al)(O,OH)_3_], jixianite [Pb(W,Fe)_2_(O,OH)_7_], elsmoreite [WO_3_.0.5H_2_O], hydrotungstite [WO_3_.2H_2_O], tungstate [WO_3_.H_2_O], anthoinite [AlWO_3_(OH)_3_], and phyllotungstite [CaFe_3_H(WO_4_)_6_.10H_2_O] [6].

Typically, the process of extracting tungsten ores involves several stages, including preconcentration, roughing, cleaning, and purification after the initial crushing and grinding. The end result of this process is usually a tungsten concentrate with a WO_3_ content of at least 65% [7]. There are various methods of reprocessing tungsten for recovery from different matrices, including gravity separation [8], magnetic separation [9], flotation [10], bioleaching [11,12], and chemical leaching [13–15]. The industrial processing of tungsten compounds involves roasting with salts like Na_2_CO_3_, NaOH, and NaNO_3_, followed by leaching with Na_2_CO_3_ or/and NaOH. Decomposition of tungsten compounds is achieved with H_2_SO_4_, HCl, and HNO_3_, forming the basis for the industrial production of tungsten [16–19].

Over the last 150 years, technology for tungsten extraction has evolved, leading to the production of sodium tungstate, metallic tungsten, and tungsten oxide. Since 1959, the industry has adopted solvent extraction, and in the 1970s, ion exchange (IX) was introduced. Recent laboratory techniques aim to enhance tungsten recovery while adhering to stringent environmental standards [20].

Various extraction methods, including solvent extraction and ion exchange, are chosen based on the composition and characteristics of the tungsten feedstock solution [21,22]. Recent attention has focused on solvent extraction with organic extractants like LIX 63 [23,24]. N1923 (primary amine, 15%) demonstrates the capability to extract tungsten from a methane-sulfonic acid/phosphoric acid mixture [25,26].

Another effective mixture involves triethyl-n-pentyl phosphonium bis-(trifluoromethylsulfonyl) amide ([P_2225_][NTf2]) and Alamine 336 for extracting tungsten from spent tungstophosphate catalyst [27]. A synergistic mixture of TRPO and TBP is utilized for extracting W from H_2_O_2_ solution [28]. The combination of TBP and P507 in a kerosene diluent is suggested as the most effective method for separating tungsten and molybdenum from iron in a solution of HCl and phosphoric acid mixture [29]. Additionally, a combination of PC 88A and LIX 63, along with other chemicals, is employed for extracting Mo(VI) and W(VI) from H_2_SO_4_ solution [30].

Furthermore, a diverse range of adsorbents and resins have been employed for successful tungsten extraction from various matrices. Lignin is utilized to create amine/quaternary ammonium lignin, which, when saturated with 1 g L^−1^ of tungsten, exhibits an impressive adsorption capability of 421.68 mg g^−1^ [31]. Polyhydroxyl chelating resin D403 is employed for studying the adsorption of vanadium and tungsten from molybdate solution, demonstrating a preference for tungsten and vanadium in batch experiments [32]. Research into ion exchange resin D314, characterized by high capacity and macroporous structure, reveals a single desorption rate approaching 90% at 60 °C, with a liquid-solid ratio of 1.25:1 and an ammonia eluent concentration of 150 g L^−1^ [33]. Specific cation exchange resin 732 facilitates rapid and increased acid leaching of tungsten from scheelite, with an overall exchange capacity of ≥4.2 mmol g^−1^ when dry [34]. Hydrotalcite, a carbonated double layer hydroxide, is utilized to successfully extract tungstate species in the form of highly charged polyoxo-metalates at a pH of around 5, employing distinct adsorption methods [35]. D309 resin is employed to preferentially adsorb tungsten over molybdenum, achieving a maximum separation factor of 9.29 at a pH value of 7 and a contact time of 4 hours [36]. Various specific resins, including D301, a very basic anion-exchange resin (type 201x7), a porous anion-exchange resin like Lewatit Monoplus MP600 (Lanxess, Germany), and anion-exchange resin AV-17-8, are all utilized for tungsten extraction [37–40].

In the course of this investigation, a novel polyvinyl chloride functionalized triethanol ammonium chloride anionite (PVC-TEAC) is fabricated and employed for tungstate recovery from Gabal Qash Amir. Located approximately 28 km southwest of Abu-Ramad city, the site is bounded by longitudes 36° 10′ 59″–36° 14′ 24″ E and latitudes 22° 14′ 07″–22° 15′ 21″ N. The newly developed PVC-TEAC is characterized using various instruments, where extraction and elution parameters are optimized for optimal performance. The investigation of tungstate extraction from its leach fluid is approached from a physico-chemical perspective, encompassing kinetics, thermodynamics, and equilibrium aspects of the process.

## 2. Experimental

### 2.1. Instrumentation

The analytical balance utilized for weighing all samples was the Sartorins TE 214S, renowned for its high precision with a maximum sensitivity of 10^−5^ g. To determine the hydrogen ion concentration, a digital pH meter from Digimed DM-21 (Japan) was employed, ensuring accuracy within an error range of ±0.1. For the equilibrium experiments, a specific weight of synthesized anionite and a fixed volume of leach liquor containing tungstate were agitated using the Vibromatic-384 shaker. To quantitatively analyze tungsten, the single beam spectrometer from Meterch Inc. (SP-8001) was employed.

The Prodigy High Dispersion ICP (ICP-OES) from TExxLEDYNE-Leeman Labs USA was employed to analyze the tungsten concentrate and establish the acceptable levels of coexisting ions. For the determination of the crystalline structure, X-ray diffraction (XRD) technique was utilized. This involved the use of a PHILIPS PW 3710/31 diffractometer, a scintillation counter, a Cu-target tube, and a Ni filter operating at 40 kV and 30 mA. To capture the infrared spectra, FT-IR 4100 Gasco-Japan spectrometer was employed with KBr disks. The ^1^H,^13^C-NMR spectra were obtained using a mercury 400 Bruker spectrometer operating at 400 MHz. The spectroscopic analysis was conducted at a temperature of 20 °C, utilizing a diluted solution with DMSO as the solvent. The chemical shift (δ) values were reported in parts per million (ppm), while the coupling constant (J) values were reported in Hertz (Hz). For the GC-MS analyses, a Shimadzu Qp-2010 Plus spectrophotometer was employed. To assess the thermal stability, thermo-gravimetric analyses (TGA) were performed under a nitrogen atmosphere using a Shimadzu TGA-50 model thermal analyzer. XPS experiments were carried out using a Kratos Axis Ultra spectrometer from Kratos, Manchester, UK, with an Al kα source emitting 225 W of monochromatic radiation. Furthermore, the elemental analysis of both the uranium concentrate product and the synthesized composites was recorded using EDX on a JSM–7900F instrument from Jeol, Tokyo, Japan.

### 2.2. Reagents

The reagents utilized in this study were prepared using high-quality analytical grade chemicals. Na_2_WO_4_.2H_2_O, Na_2_MoO_4_.2H_2_O, malachite green (MG), and Sintanol ALM-10 nonionic surfactant were sourced from Merck, Germany. PVC, HCl, H_2_SO_4_, and HNO_3_ analytical grade reagents were obtained from POCH S.A., Poland. Ethylene diamine dihydrochloride, chloromethyl methyl ether (CMME), and triethanol amine were procured from Scharlau Chemie. S.A., Spain. Methanol, ethyl acetate, and DMF were also purchased from Scharlau Chemie S.A., Spain.

### 2.3. Preparation of a stock of standard solutions

A standardized stock solution containing 1000 mg L^−1^ (5.4 × 10^−3^ M) of W(VI) was meticulously prepared. This was achieved by dissolving a precise weight of 1.794 g of Na_2_WO_4_.2H_2_O in 1000 mL of distilled water, along with the addition of 2 mL of 30% sodium hydroxide to prevent any potential hydrolysis. In contrast, numerous standard stock solutions, each with a concentration of 1000 mg L^−1^, were also prepared for various ions that could potentially be involved in the adsorption of W(VI) by PVC-TEAC anionite. These solutions were obtained by dissolving the appropriate weight of their respective salts in 1000 mL of distilled water. The purpose of these preparations is to ensure accurate and reliable experimentation in the investigation of W(VI) adsorption.

### 2.4. Batch-static adsorption procedures

The optimization of factors influencing the adsorption of W(VI) from a synthetic solution by PVC-TEAC anionite using a batch-static technique was investigated. Various parameters, including pH, contact time, initial W(VI) concentration, anionite dose, temperature, and diverse ions, were considered in these experiments. In each experiment, 25 mL of synthetic W(VI) solution with a concentration of 150 mg L^−1^ (0.81×10^−3^ M) W(VI) was mechanically shaken at 300 rpm with 0.05 g of anionite for a specific duration at different temperatures. The uptake capacity of W(VI) (q_e_), expressed in mg g^−1^, was calculated using Equation 1 [41].


(1) $$q_{e}=(C_{0}-C_{e})\times \frac{V}{m},$$

In this equation, V is the volume of the aqueous solution (L) containing W(VI), m is the dry composite weight (g), and C_o_ and C_e_ are the initial and equilibrium W(VI) concentrations (mg L^−1^) correspondingly. Meanwhile, Equation 2 can be used to determine the distribution coefficient (K_d_), where V is the volume of the aqueous phase in L [42]:


(2) $$K_{d}=\frac{C_{0}-C_{e}}{C_{0}}\times \frac{V}{m},$$

### 2.5. Batch-static elution procedures

Various eluting agents were investigated for their effectiveness in reextracting W(VI) from PVC-TEAC anionite. To conduct the experiments, 0.05 g of loaded anionite was mixed with 10 mL of each eluting agent at varying concentrations. The mixture was then shaken for 10 min at room temperature. The objective was to determine the optimal eluting agent for W(VI) elution procedures.

### 2.6. Analytical procedures for W(VI) analysis

In this study, W(VI) was examined using a single beam spectrophotometer, Meterch (SP-8001) with malachite green indicator (MG) in various aqueous phases. To ensure accuracy, Sintanol ALM-10 nonionic surfactant was used, and measurements were taken at a wave length of 620 nm against a proper reagent blank. The results showed that the calibration graph followed Beer’s law within the concentration range of 1 × 10^−6^ to 1 × 10^−5^ mol L^−1^ W(VI). The equation A = (7.06 ± 0.43) × 10^4^ × CW was found to be the best fit, where CW is the concentration of W(VI), mol L^−1^. The correlation coefficient was calculated to be 0.997, and the molar absorption coefficient was equal to 7.06 × 10^4^ L mol^−1^ cm^−1^ [43].

## 3. Results and discussion

### 3.1. Synthesis of polyvinyl chloride functionalized triethanol ammonium chloride anionite (PVC-TEAC)

PVC-TEAC, or polyvinyl chloride functionalized triethanol ammonium chloride anionite, is synthesized through a meticulously executed four-step process. In the first step, initial neutralization step, the process commences by refluxing a mixture of 0.16 moles (approximately 21.28 g) of ethylene diamine dihydrochloride with 0.16 moles of NaOH (approximately 6.4 g) in 50 mL of absolute ethanol as a diluent. This gentle refluxing at 25 °C for 2 h enhances the nucleophilicity of ethylene diamine towards PVC. The resulting white precipitate is obtained by turning off the condensation reaction, cooled to room temperature, washed three times with 100% ethanol using a vacuum air Buchner and then dried at 50 °C for 3 h. In the second nucleophilic substitution step, the neutralized ethylene diamine dihydrochloride is added to 0.16 moles of polyvinyl chloride (10 g) and 0.16 moles of NaOH (6.4 g) in 50 mL of DMF as a diluent. This mixture undergoes a 4-h condensation in a condenser at 110 °C, forming the PVC-en composite. In the third nucleophilic substitution step, the PVC-en composite (10 g, approximately 0.116 moles) is condensed with 0.116 moles (approximately 8.8 mL) of chloromethyl methyl ether (CMME) in 50 mL of DMF as a diluent. This 5-h process at 110 °C introduces the chloromethyl group to PVC-en composite, resulting in PVC-en-MC composite. In the final nucleophilic substitution step, the PVC-en-MC composite (10 g, approximately 0.074 moles) is condensed with 0.074 moles (approximately 11 mL) of triethanol amine in a condenser for 5 h at 110 °C, culminating in the formation of the final product, PVC-TEAC.

**Figure 1:**
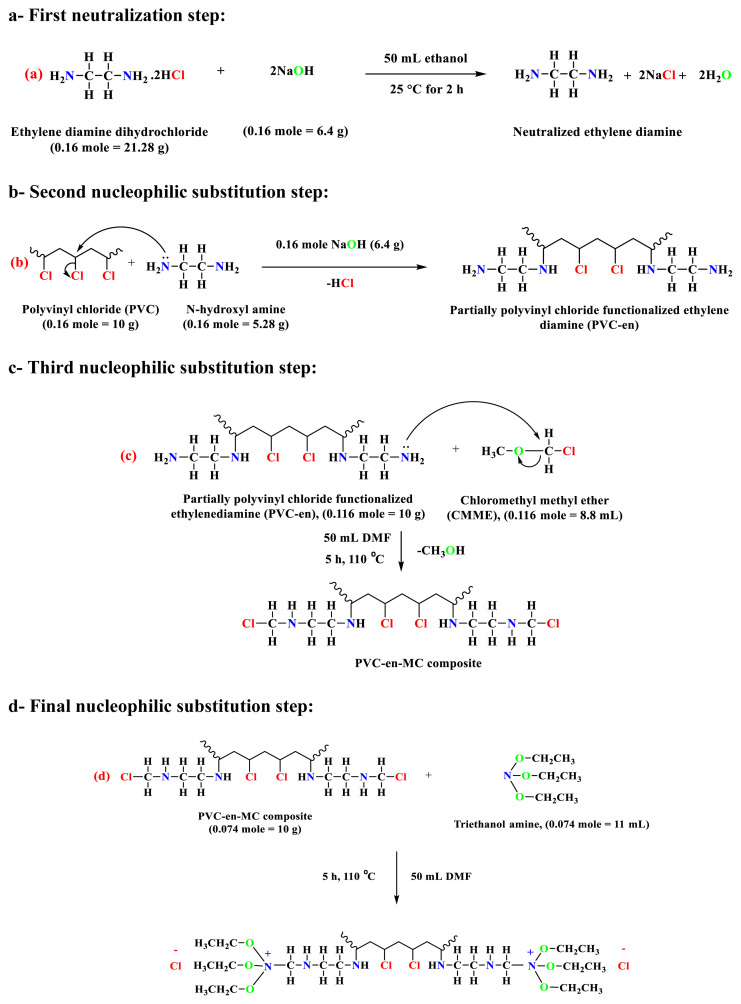
Synthesis of partially polyvinyl chloride functionalized triethanol ammonium chloride anionite (PVC-TEAC). a- First neutralization step, b- second nucleophilic substitution step, c- third nucleophilic substitution step, d- final nucleophilic substitution step.

**Figure 2:**
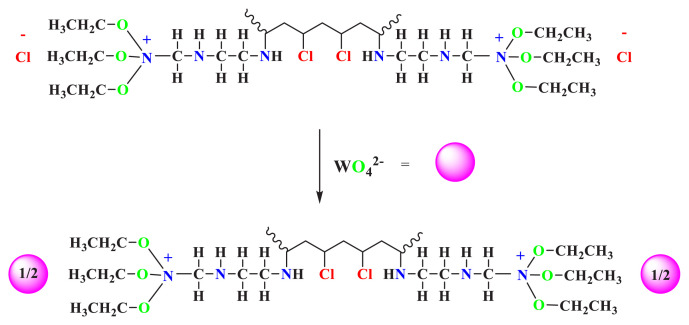
A proposed mechanism for W(VI) exchange by PVC-TEAC anionite.

Following the completion of the reaction, the product is obtained by cooling and thorough washing with 100% ethanol to eliminate any residual DMF and undesired by-products. The resulting precipitate is then subjected to 3 h of drying at 110 °C, finalizing the production of PVC-TEAC anionite, which boasts a density of approximately 1.23 g cm^−3^. Figure 1 illustrates the synthesis of PVC-TEAC, along with the proposed reaction mechanism. Figure 2 presents a conceptual illustration of the potential adsorption mechanism for W(VI) by PVC-TEAC anionite.

### 3.2. Specification of polyvinyl chloride functionalized triethanol ammonium chloride anionite (PVC-TEAC)

The product yield was found to be ≈ 13 g; **m.p** ≈ 265–270 °C; **FT-IR (KBr) v/cm**^−^**^1^**: 2846, 2908, 2962 (-CH aliphatic), 1331, 1427 (-N-O), 1242 (-C-N), 3441 (-NH), 486, 416 (-C-Cl) and 840, 964, 694 (-W-O). **^1^H-NMR (400.16 MHz, DMSO-*d*_6_, 25 °C, TMS) δ, ppm**: 1.71 (t, 2H, (-CH_2_)_n_, J = 6.93 Hz), 4.1 (m, 1H, (-CHCl)_n_, J = 6.93 Hz), 3.29 (m, 1H, (-CH-NH-)_n_, J = 7.31 Hz), 4.09 (m, 1H, -NH, J = 6.33 Hz), 3.76 (m, 2H,-OCH_2_CH_3_, J = 7.32 Hz), 1.3 (t, 3H, -OCH_2_CH_3_, J = 7.32 Hz). **^13^C-NMR (100.06 MHz, DMSO-*d*_6_, 25 °C, TMS) δ, ppm**: 55.45 (s, -CH-Cl, J = 3.3 Hz), 45.75 (s, (-CH_2_)_n_, J = 3.3 Hz), 50.89 (s, -CH-NH-), 55.53 (s, -CH-N-), 62.86 (s, -OCH_2_CH_3_, J = 3.7 Hz), 15.4 (s, -OCH_2_CH_3_, J = 3.7 Hz). **GC-MS (EI, 15 eV), m/z (% rel): [m/z]^+^** : 283, 285, 287, 178, 158, 128, 93, 78, 62, 43, 36, 29, 17, 15. **Anal. Calc. for [C_11_H_26_N_3_O_3_Cl]_n=1_** building unit (283.45 g/mol): **C**, 46.57 ; **H**, 9.17 ; **N**, 14.81 ;**O**, 16.93; **Cl**, 12.5. **Found**: **C**, 46.55; **H**, 9.2 ; **N**, 14.78 ;**O**, 17; **Cl**, 12.45.

#### 3.2.1. Infrared analysis (FT-IR)

**Figure 3:**
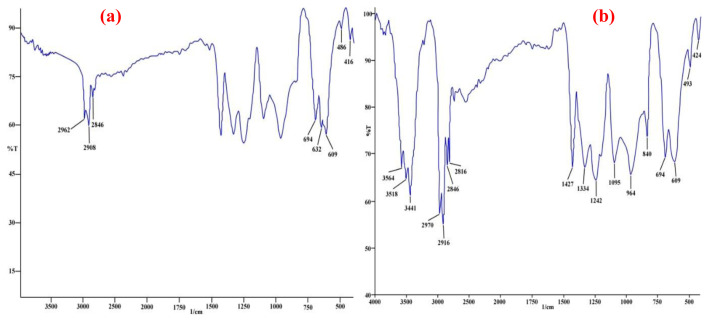
FT-IR spectra of (a) unmodified PVC, (b) PVC-TEAC-W.

The Fourier transformation infrared spectroscopy (FT-IR) technique was employed to analyze the synthesized PVC-TEAC anionite, leading to significant findings. Various functional groups in the PVC were identified, including -CH aliphatic groups at 2846, 2816, 2916, and 2970 cm^−1^, as well as -C-Cl groups at 493, 424, 609, and 694 cm^−1^. Upon the preparation of PVC-TEAC, new assignments were observed, such as -NH groups at 3441, 3518, and 3564 cm^−1^, -C-N groups at 1242 cm^−1^, and -N-O groups at 1334 cm^−1^ (sym.) and 1427 cm^−1^ (asym.). Notably, a shift to lower frequencies of approximately 10–15 cm^−1^ was observed for the -NH, -N-O, and -C-N groups. However, the other assignments in PVC-TEAC remained unchanged after adsorption with W(VI), including the -CH aliphatic groups at 2846, 2816, 2916, and 2970 cm^−1^, as well as the -C-Cl groups at 493, 424, 609, and 694 cm^−1^. Furthermore, the appearance of new bands at 840, 964, and 1095 cm^−1^ in the composite suggests the formation of a coordinated O-W-O bond [44,45]. The FT-IR spectra of (a) unmodified PVC (b) PVC-TEAC with W(VI) are depicted in Figure 3.

#### 3.2.2. X-ray photoelectron spectroscopy analysis (XPS)

**Figure 4:**
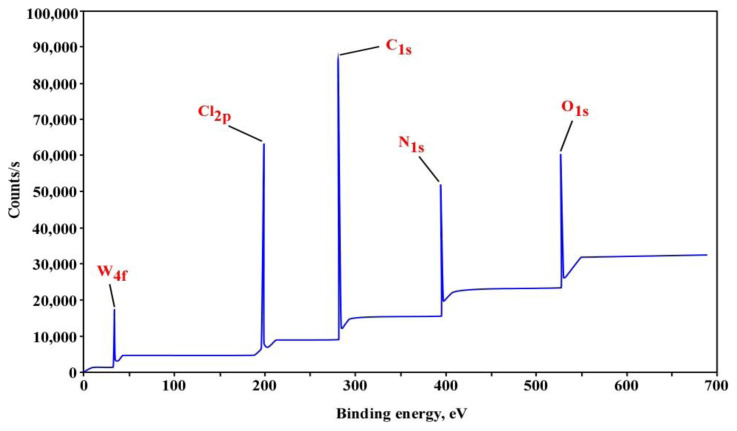
XPS spectra of PVC-TEAC-W anionite.

The chemical compositions of unmodified PVC, PVC-TEAC, and PVC-TEAC-W were investigated using X-ray photoelectron spectroscopy (XPS), and the results are depicted in Figure 4 and summarized in Table 1. All three samples exhibited peaks corresponding to C*_1S_* and Cl*_2P_* at 287 eV (carbon content: 38%) and 199.5 eV (chlorine content: 56.37%), respectively [46]. In comparison to PVC, PVC-TEAC demonstrated an increase in carbon content to 47.57%, the emergence of new peaks at 398 and 530 eV attributed to N*_1S_* and O*_1S_*, respectively [47], and a significant reduction in chlorine content from 56.37% to 15.6%. This reduction indicates the successful condensation of PVC to PVC-TEAC, with an immobilizing efficiency of 72.32%. Furthermore, PVC-TEAC exhibited higher nitrogen and oxygen contents of 17.31% and 19.5%, respectively, compared to PVC, indicating effective immobilization.

Following the successful immobilization of PVC, the exchange of W(VI) by PVC-TEAC to form the tungsten composite was confirmed by a distinctive peak at 35.6 eV, corresponding to the presence of W*_4f7/2_*, with a tungsten content of 1.25% [48]. In conclusion, the conversion of PVC into PVC-TEAC anionite through chemical functionalization proves to be a successful and fruitful technique.

#### 3.2.3. Brunauer–Emmett–Teller (BET) analysis

The Brunauer–Emmett–Teller (BET) method was employed to analyze N_2_ sorption-desorption on PVC, PVC-TEAC, and PVC-TEAC-W. Table 2 presents the specific pore volume (V, cm^3^ g^−1^), surface area (S_BET_, m^2^ g^−1^), and the average pore diameter (d, nm) for each material. For unmodified PVC, the specific pore volume, surface area, and average pore diameter were measured at 0.0262 cm^3^ g^−1^, 24.75 m^2^ g^−1^, and 1.97 nm, respectively [49]. Following the modification to create PVC-TEAC, significant enhancements were observed in the pore volume (0.0935 cm^3^ g^−1^), surface area (45.14 m^2^ g^−1^), and pore diameter (2.1 nm).

Subsequently, the exchange mechanism between PVC-TEAC and W(VI) led to the formation of the PVC-TEAC-W composite, resulting in a reduction in specific pore volume (0.0889 cm^3^ g^−1^), surface area (43.068 m^2^ g^−1^), and pore diameter (2 nm) compared to PVC-TEAC anionite. The average pore diameter of 2.1 nm for PVC-TEAC and 2 nm for PVC-TEAC-W indicated that they both fall within the microporous scale. These findings validate the successful modification of PVC to PVC-TEAC and the subsequent chelation to form the PVC-TEAC-W composite. The surface area (S_BET_), specific pore volume (V), and the average pore diameter (d) for PVC, PVC-TEAC, and PVC-TEAC-W are summarized in Table 2 and visualized in Figure S1.

#### 3.2.4. SEM-EDX analysis

Surface morphology examination at 3000× magnification, with a 5-μm full scale and an energy of 15 Kev, was conducted using SEM to analyze PVC, PVC-TEAC, and PVC-TEAC-W. The observations revealed that PVC exhibited a microstructure that was exceptionally smooth and polished. The surface morphology underwent a noticeable transformation, becoming more uneven, after the condensation of PVC to form PVC-TEAC. This transformation was a result of the alteration in surface morphology.

**Figure 5:**
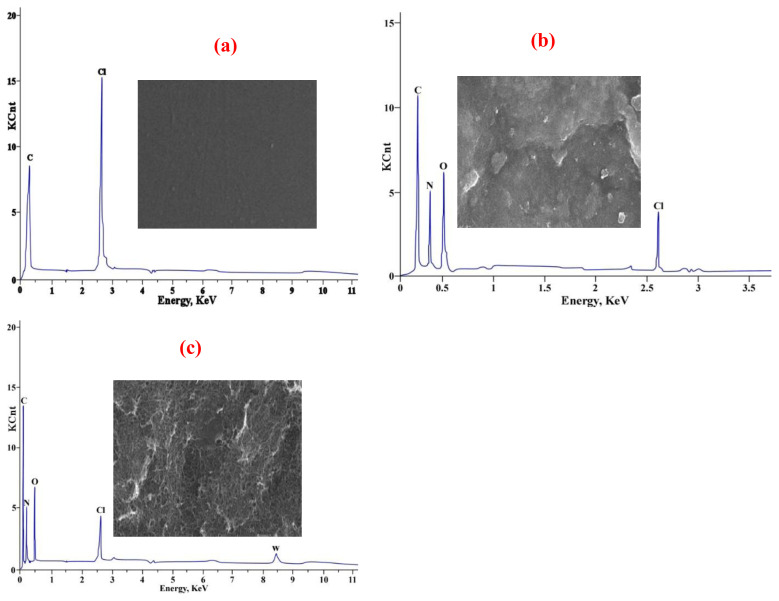
SEM-EDX of (a) PVC, (b) PVC-TEAC, (c) PVC-TEAC-W.

Upon the introduction of W(VI) ions to PVC-TEAC, the surface underwent further transformation, displaying a more uneven, sinuous networking microstructure, and rough appearance. This change was attributed to the adsorption of W(VI) ions into the surface. SEM-EDX analysis confirmed the chemical composition of PVC, PVC-TEAC, and PVC-TEAC-W, revealing the presence of C and Cl for PVC, C, Cl, O, and N for PVC-TEAC, and C, Cl, O, N, and W for PVC-TEAC-W. These findings were consistent with the ability of PVC-TEAC anionite to adsorb W(VI) ions, affirming the success of its manufacture from PVC. The SEM-EDX of free PVC (Figure 5a), modified PVC (PVC-TEAC) (Figure 5b), and loaded PVC-TEAC-W (Figure 5c) were illustrated.

#### 3.2.5. TGA analysis

**Figure 6:**
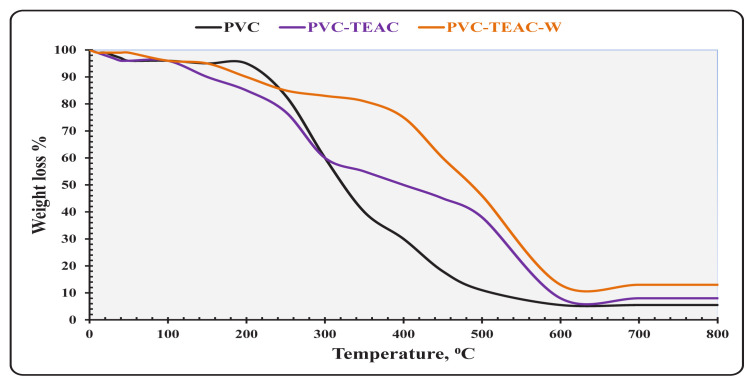
Thermogram of PVC, PVC-TEAC, and PVC-TEAC-W in N_2_ environment.

Thermogravimetric analysis (TGA) was employed to assess the impact of chemical alterations on the thermal stability of PVC composites. Figure 6 presents the TGA thermographs of PVC, PVC-TEAC, and PVC-TEAC-W, ranging from ambient temperature to 800 °C, with a heating rate of 15 °C min^−1^. The TGA steps, deflection temperature, weight loss percentage, and the subsequent weight residue are summarized in Table 3.

According to the TGA data, all samples, including PVC, PVC-TEAC, and PVC-TEAC-W, experienced a weight loss of approximately 5% around 100 °C, attributed to the evaporation of physically weak and chemically strongly bound water [50]. Beyond 100 °C, notable differences emerged between PVC and the other samples. For PVC, three distinct decomposition steps were observed: 100–250 °C (weight loss 13%), 250–450 °C (weight loss 64%), and 450–600 °C (weight loss 12.5%), with a final char residue of 5.5% at 600–800 °C. The substantial weight loss at 250–450 °C may be attributed to side chain polyene and cyclization reactions (dehydrochlorination of PVC), with the release of HCl from the PVC polymer chain. The temperature range of 450–600 °C (weight loss 12.5%) may be associated with carbonization and breakdown processes, while the third phase may relate to thermo-oxidation [51].

Similar to PVC, PVC-TEAC displayed four distinct steps: 100–265 °C (weight loss 19%), 265–500 °C (weight loss 40%), 500–600 °C (weight loss 30%), and the appearance of a final weight residue stage with 8% weight loss at 600–800 °C. This final stage may be attributed to the increased organic components, representing ethylene diamine and triethanol amine.

PVC-TEAC-W also exhibited four stages: 100–345 °C (weight loss 15%), 345–500 °C (weight loss 36%), 500–600 °C (weight loss 33%), and a final high weight char stage with a 13% weight loss, indicative of the formation of residual tungsten oxide with char residue. The shift to a higher degradation temperature at 265 °C for PVC-TEAC (higher than PVC), and further to 345 °C for PVC-TEAC-W (higher than PVC-TEAC), suggests the successful adsorption of W(VI) ions onto PVC-TEAC, enhancing the thermal properties [52].

#### 3.2.6. ^1^H-NMR analysis

**Figure 7:**
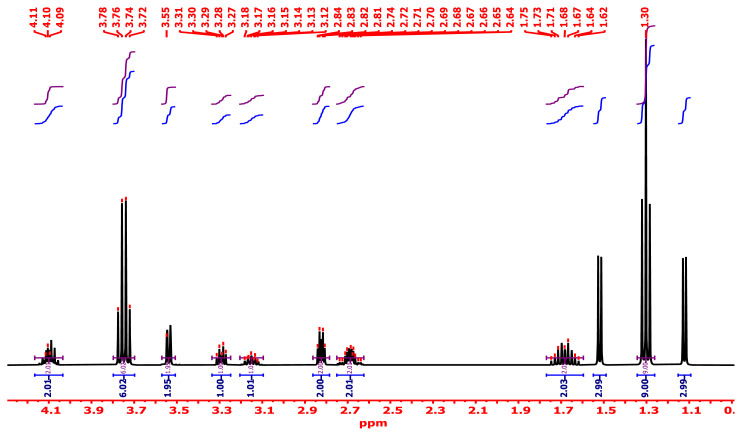
Specification of PVC-TEAC anionite by ^1^H-NMR spectrometry.

^1^H-NMR analysis with an energy of 400.16 MHz, DMSO-*d_6_* as a diluent, and tetramethyl silane as a certified sample at 25 °C is an excellent instrument that delivers substantial data regarding protons in the synthesized PVC-TEAC anionite, which aid in the structure suggested. The main δ (ppm) assignments for the unmodified PVC main skeleton appeared at 1.71 and 4.1, which were related to the −(CH_2_)_n_ and −(CH)_n_-Cl groups, respectively. It was discovered that the methine proton, which was directly linked to the chlorine atom (an electronegative atom), is more deshielded than the typical methylene group, which caused an increase in the value of its chemical shift. The other chief δ (ppm) assignments for the branched PVC-TEAC appeared at 3.15, 4.09, and 2.82 ppm, which were associated with methine groups attached to nitrogen (−CH-N-), nitrogen proton (−NH), and methylene protons flanked between two nitrogen atoms. It was hypothesized that the fact that various protons have more than one assignment indicates the asymmetry of the composite that is caused by the functionalization. It was discovered that the branch of the triethanol amine moiety produces different assignments at 3.76 and 1.3 ppm, which respectively represent the methylene and methyl protons of the ethoxide moiety (−OCH_2_CH_3_). Figure 7 provides an illustration of the PVC-TEAC composite’s specifications as determined by ^1^H-NMR.

#### 3.2.7. ^13^C-NMR analysis

**Figure 8:**
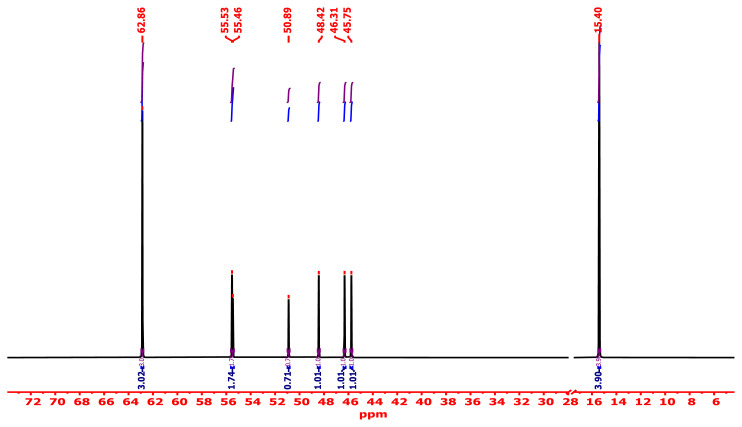
Specification of PVC-TEAC anionite by ^13^C-NMR spectrometry.

The ^13^C-NMR study, performed with an energy of 100.04 MHz and DMSO-*d_6_* as a diluent, is a useful method that provides considerable data about the amount of carbon atoms in the synthesized PVC-TEAC anionite. The main skeleton of the remaining unmodified PVC has the main δ (ppm) appearing at 55.45 and 45.75 ppm as singlet which were related to −CH-Cl carbon and methylene group (−CH_2_) _n_. The high value of the chemical shift of −CH-Cl carbon may be due to the attachment of the more electronegative chlorine atom. The other main δ (ppm) assignments for the branched PVC-TEAC appeared at 50.89, 48.42, and 55.53 ppm, which were related to methine carbon attached to nitrogen (−CH-NH-), methylene carbon of the ethylene diamine moiety, and methylene carbon attached to the quaternary nitrogen. It was found that the branch of the triethanol amine moiety gives a distinct assignment at 62.86 and 15.4 ppm, which represent methylene and methyl carbon of the ethoxide moiety (−OCH_2_CH_3_), respectively. The specification of the PVC-TEAC composite using ^13^C-NMR is shown in Figure 8.

#### 3.2.8. Mass analysis

To predict the presence of the base peak (associated with the more stable fragment) and the quasimolecular ion peak (related to the molecular formula), a mass spectrometer that includes a gas chromatography unit (GC-MS) is typically employed. The molecular formula of the synthesized PVC-TEAC is [C_11_H_26_N_3_O_3_Cl]_n=1_, and it is represented by the quasimolecular ion peak with a value of 283 and a relative abundance of 37%. Several important fragmentation patterns, which are associated with the newly manufactured PVC-TEAC anionite, were identified. These patterns include: [C_2_H_3_Cl]_n=1_^•^ with m/z = 62 and a relative abundance of 23%, which is related to the vinyl chloride moiety, [C_2_H_5_N]_n=1_^•^ with m/z = 43 and a relative abundance of 27%, which is related to vinyl amine moiety, which represents good amination to PVC. Additionally, these findings confirm the existence of residual vinyl chloride building blocks in the PVC-TEAC anionite chain. In addition to this, additional fragments that are regarded as indicators of the effective production of PVC-TEAC, such as [NH_3_] ^•^ with m/z = 17 and a relative abundance of 22%, which is related to ammonia gas, [HCl] ^•^ with m/z = 36 and a relative abundance of 78%, which is related to HCl gas. The fragmentation of the branched chain of the PVC-TEAC moiety led to the formation of methyl and ethyl radicals, which were detected at [CH_3_] ^•^ with m/z = 15 and a relative abundance of 5% and [C_2_H_5_] ^•^ with m/z = 29 and a relative abundance of 9%, respectively. The mechanism of fragmentation patterns in PVC-TEAC anionite should be noted because it contains chlorine atoms. As the number of chlorine atoms increases, the quasimolecular ion peak (M = 283, relative abundance 37%) and the M+2 and M+4 isotopic peaks (M = 285, relative abundance 11%) become more prominent. If there is more than one chlorine atom in the molecule, a M+4 isotopic peak with a relative abundance of 17 should be seen at m/z = 287.

**Figure 9:**
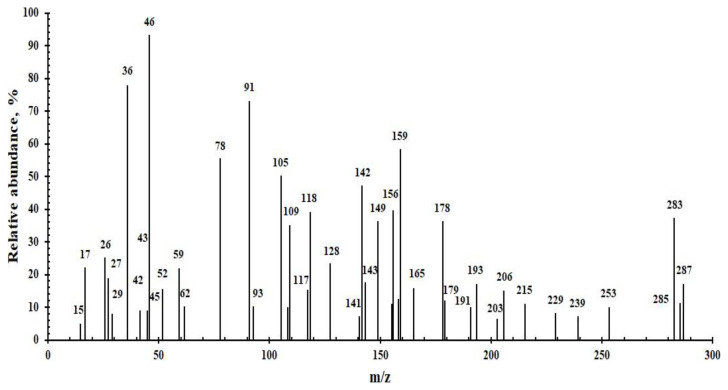
Specification of PVC-TEAC anionite by mass analyzer.

It is common knowledge that when PVC-TEAC is subjected to an electron flux, HCl (m/z = 36), with a relative abundance of 78%, is released, resulting in the formation of polymerized polyene, substituted polyenes, aromatics, and condensed aromatics, which can then combine to form cyclic compounds such as benzene [C_6_H_6_] ^•^ with m/z = 78 and a relative abundance of 55%, aniline [C_6_H_7_N] ^•^with m/z = 93 and a relative abundance of 10%, naphthalene [C_10_H_8_] ^•^with m/z = 128 and a relative abundance of 23%, anthracene [C_14_H_10_] ^•^ with m/z = 178 and a relative abundance of 36% and diamino naphthalene [C_10_H_10_N] ^•^with m/z = 158 and a relative abundance of 12%. It is evident from the analysis that the PVC-TEAC composite can be successfully synthesized. Figure 9 represents a GC-MS depiction of a PVC-TEAC description.

### 3.3. Adsorption features

#### 3.3.1. The effect of pH

**Figure 10:**
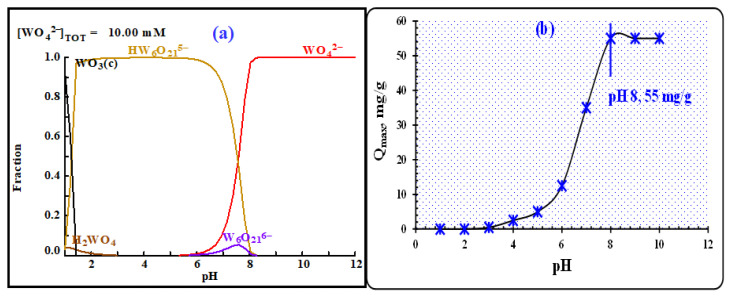
(a) Speciation-distribution diagram of W(VI) ions in solution at various pH levels, (b) the effect of pH on W(VI) retention by PVC-TEAC anionite. *(Conditions: V: 25 mL, W(VI) conc.: 150 mg L*^−^*^1^, m: 0.05 g, T: 25 °C, agitation time : 5 min)*

The retention of W(VI) in all composites is significantly influenced by pH, as it alters both the solution chemistry of tungsten and the characteristics of the active sites in composites. This interaction between these factors plays a crucial role. Figure 10a depicts the speciation of tungstate ions at various pH values using the HYDRA-MEDUSA program. Tungsten’s solution chemistry is essential, with tungsten commonly existing as tungstic acid (H_2_WO_4_, at pH ≤ 2), poly-tungstate (HW_6_O_21_^5−^, W_6_O_21_^6−^, at pH 2–7), and tungstate (WO_4_^2−^, at pH 7–12), depending on the concentration of tungsten in the solution and the pH [6].

To study the retention of W(VI) on PVC-TEAC anionite in the pH range from 1 to 10, 25 mL of a 150 mg L^−1^ W(VI) solution, and 0.05 g of anionite were used at room temperature within 5 min. Figure 10b presents the results, showing virtually no retention at extremely acidic conditions (pH 1–3) due to the presence of tungstic acid (H_2_WO_4_↓) and polynuclear tungstate (HW_6_O_21_^5−^), which was not exchanged with the counter chloride anion of PVC-TEAC anionite. As the pH increased from 3 to 8, the formation of mononuclear tungstate (WO_4_^2−^) occurred, leading to an increase in the retention of W(VI) and a subsequent rise in the overall retention. The highest point was reached at pH 8 (Q_max_ = 55 mg g^−1^), and retention returned to its original value at pH 10, where it remained unchanged. Therefore, pH 8 proved to be the most effective pH for W(VI) retention on PVC-TEAC anionite.

#### 3.3.2. The effect of time of agitation

**Figure 11:**
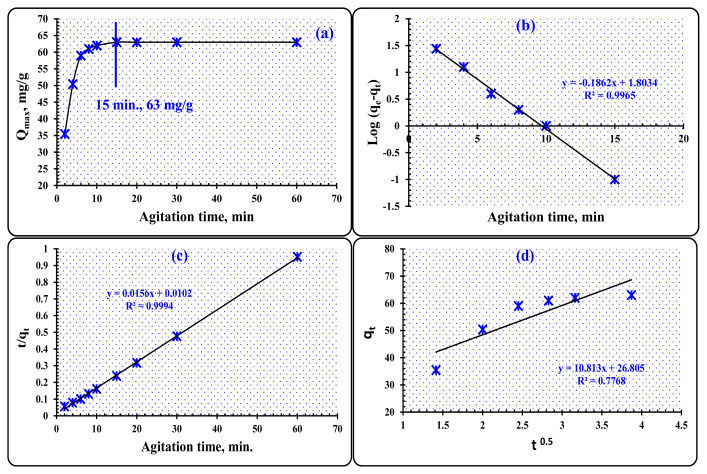
(a) Effect of agitation time on W(VI) ions uptake by PVC-TEAC anionite, (b) pseudofirst-order modeling, (c) pseudosecond-order modeling, (d) intraparticle diffusion modeling. *(Conditions:* *V: 25 mL, W(VI) conc.: 150 mg L*^−^*^1^, m: 0.05 g, T: 25 °C, pH: 8).*

In terms of cost, one of the most critical factors to consider is the duration of agitation. The impact of agitation duration on the capture of W(VI) ions was investigated over a range of 2 to 60 min using 0.05 g of PVC-TEAC anionite and 25 mL of a tungsten aqueous solution with a concentration of 0.81 × 10^−3^ mol L^−1^ (150 mg L^−1^) at a pH of 8. Figure 11a presents the findings, demonstrating that the maximum adsorption of W(VI) ions increases with an increasing duration of agitation time, reaching its peak value at 15 min (63 mg g^−1^, 84% efficiency), and then remaining virtually constant until 60 min. The results were exhibited and illustrates that the maximum uptake of W(VI) ions increases with an increasing length of agitation time. Consequently, 15 min proved to be more than sufficient for reaching equilibrium in future testing, and this time duration was used in all of the remaining studies, indicating that appropriate kinetics was attained.

##### 3.3.2.1. Kinetic prospects

The rate of adsorption may be described by studying the kinetics of the absorption of W(VI) ions on PVC-TEAC anionite. The kinetic parameters provide essential data for designing and modelling adsorption processes as well as helping to determine the predictions of the adsorption rate. Using pseudofirst-order, second-order, and intraparticulate diffusion models, we were able to predict the rate constants of the uptake process as well as the proposed mechanism for the W(VI) ions adsorption upon PVC-TEAC anionite. Below is a calculated equation that can be used to define the pseudofirst-order kinetic model [53].


(3) $$Log(q_{e}-q_{t})=Log\;q_{e}-\left(\frac{K_{1}}{2.303}\right)\;t,$$

where K_1_ (min^−1^) signifies a constant rate, q_e_ is the quantity of W(VI) ions adsorbed in a balanced way per unit mass (t, min^−1^). Figure 11b shows a straight line, the slope and intercept of which give the first-order adsorption rate constants K_1_ and q_e_. These values may be derived by plotting Log (q_e_-q_t_) versus agitation time, t. The calculated q_e_ values were 63.6 mg g^−1^, which is close to the true value (63 mg g^−1^), and the rate of adsorption was (K_1_ = 0.428 min.^−1^, R^2^ = 0.9965). Providing a satisfactory explanation for the observed phenomena, the plot diagram below suggests that pseudofirst-order kinetic modelling could be applied. The following equation defines pseudosecond-order kinetic modelling [54]:


(4) $$\frac{t}{q_{t}}=\frac{1}{K_{2}\;q_{e}^{2}}+\frac{t}{q_{e}},$$

Here, K_2_ represents the steady rate (g/mg min.). The intercept on the t/q_t_ line is equal to 1/k_2_q_e_^2^, and the slope is 1/q_e_. Pseudosecond-order kinetic modelling was demonstrated to be applicable to experimental data in Figure 11c. Using a correlation coefficient of R^2^ = 0.9994 and an adsorption rate of (K_2_ = 0.0102 g mg^−1^ min^−1^), the authors calculated a value of q_e_ of 64.1 mg g^−1^, which was very close to the value of 63 mg/g observed in practice. The findings showed that the kinetic model of second-order is likewise consistent with the data of the experiments, and as a consequence, it is suitable for use in attempting to describe the system that is the subject of the study. According to the findings, the first-order modelling and the second-order modelling may be considered mixed models for the purpose of successfully interpreting the adsorption system.

In the context of the adsorption of W(VI) onto PVC-TEAC anionite, the interaction between a liquid and a solid plays a crucial role. The rate of W(VI) ion adsorption is influenced by various mechanisms that occur at the liquid-solid interface, such as (1) bulk diffusion mechanism, which involves diffusion from the bulk of the solution to the film around the active sites of the composite, (2) external diffusion mechanism, at which intersphere diffusion of W(VI) ions occurs in this case, affecting the overall adsorption rate, (3) intraparticle diffusion mechanism, which involves diffusion within a particle or pore, influencing the adsorption process, (4) physical and chemical adsorption, ion exchange, and complexation mechanisms are only a few of the various routes through which direct contact between W(VI) ions and PVC-TEAC anionite sites may occur [55].

The rate of adsorption is impacted by the external diffusion mechanism, where the agitation speed of the system controls the thickness of the layer at the liquid-solid interface. An increase in agitation speed results in a reduction of this layer’s thickness.

To assess the rate-controlling step in the adsorption of W(VI) ions on PVC-TEAC anionite, the intraparticle diffusion model equation proposed by Weber and Morris is applied. This involves calculating the square root of the equilibrium time, which is a crucial parameter for understanding the rate-controlling stage in the adsorption process [56].


(5) $$q_{t}=K_{ad}\sqrt{t}+I,$$

K_ad_ is the rate constant (mg g^−1^ min^1/2^), I is the boundary layer thickness, and q_t_ is the absorption capacity of W(VI) ions at time t. The rate constant can be calculated from the plot of linear gradients of q_t_ against t^1/2^ (Figure 11d). The calculated boundary layer thickness (I) was 26.8, the constant intraparticle diffusion rate (K_ad_) was 10.813 mg g^−1^ min^1/2^, and the R^2^ correlation coefficient was found to be 0.7768. The low value of the correlation coefficient prevents its application in the interpretation, but the positive value of (I) shows that the intraparticle diffusion mechanism may regulate W(VI) ions adsorption upon PVC-TEAC anionite. Adsorption kinetics characteristics for W(VI) on PVC-TEAC anionite are shown in Table 4.

#### 3.3.3. The effect of initial concentration of tungsten ions

**Figure 12:**
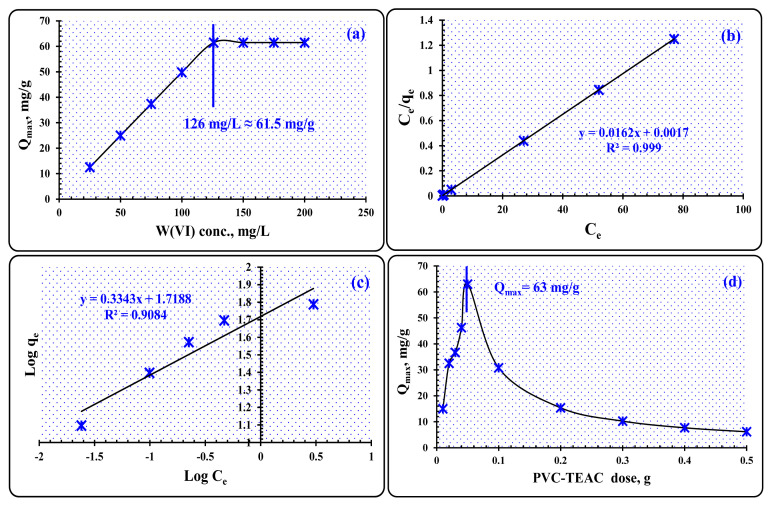
(a) Effect of initial W(VI) ions concentration on tungsten uptake by PVC-TEAC, (b) Langmuir isotherm, (c) Freundlich isotherm, (d) effect of PVC-TEAC dose on W(VI) uptake. *(Conditions: V: 25 mL, W(VI) conc.: 150 mg L*^−^*^1^, m: 0.05 g, T: 25 °C, pH 8, Agitation time : 15 min).*

In summary, the investigation of the relationship between the uptake of W(VI) ions and the initial tungsten concentration reveals two distinct stages, as illustrated in Figure 12a: in the first stage (25 to 126 mg L^−1^), which is characterized by a remarkable increase in tungsten absorption. The rise is attributed to the saturation of PVC-TEAC anionite by W(VI) ions, which may not be achieved at low concentrations of tungsten. The quantity of W(VI) ions is relatively low compared to the number of active sites in the PVC-TEAC composite during this stage.

In the second stage (126 to 200 mg L^−1^), once the saturation of PVC-TEAC active sites with W(VI) ions is reached, the absorption of tungsten ions remains relatively constant. Concentrations ranging from 126 to 200 mg L^−1^ demonstrate a plateau in the absorption of tungsten ions. At room temperature and a tungsten concentration of 126 mg L^−1^, the tungsten absorption reaches its highest point at 63 mg g^−1^.

These observations suggest that there is an optimal range of tungsten concentrations for efficient adsorption by PVC-TEAC anionite. Beyond a certain concentration, the active sites become saturated, leading to a steady-state in tungsten absorption. Understanding these concentration-dependent dynamics is crucial for optimizing the adsorption process.

##### 3.3.3.1. Distribution-isotherm modeling

The number of adsorbed W(VI) ions on the PVC-TEAC composite was calculated to calculate the equilibrium W(VI) ion concentration at room temperature. The Langmuir technique relies on the three assumptions that (a) maximum adsorption corresponds to a monolayer of adsorbate molecules on the adsorbent surface, (b) the energy needed for adsorption is constant, and (c) no adsorb transmigration happens on the surface plane. These three assumptions are what allow the Langmuir treatment to work. The computed equation provides a way to derive the model of the Langmuir isotherm [57,58]:


(6) $$\frac{C_{e}}{q_{e}}=\frac{1}{q_{e}b}+\frac{C_{e}}{q_{e}},$$

where C_e_ is the equilibrium concentration in milligrams per milliliter, q_e_ is the amount of W(VI) ions bound at equilibrium, and q_e_ and b are Langmuir constants pertaining to the maximum adsorption potential in milligrams per game and the adsorption energy in milliliters per milligram, respectively. Figure 12b displays a linear relationship between C_e_/q_e_ and C_e_, which can be used to establish the Langmuir model. This curve demonstrates that the adsorption procedure is consistent with the Langmuir model. It was established that the correlation coefficient for the linear regression that corresponds to the Langmuir plot is equal to 0.999 for the R^2^ value. The slope and the intercept were used to compute q_e_ and b, and they came out to be 61.728 mg g^−1^ and 9.523 g mg^−1^, respectively. The results may be seen in the table below. The value of the computed q_e_ is much closer to the amount that was obtained via experimentation (63 mg g^−1^). The main features of the Langmuir isotherm can be described using a dimensionless separation factor, sometimes called an equilibrium parameter, R_L_. The equation used to derive the isotherm provides the value for this parameter [59]:


(7) $$R_{L}=\frac{1}{1+bC_{0}},$$

where b is the Langmuir constant and C_o_ is the concentration of W(VI) ions at the outset, which can be anywhere from 25 to 200 mg L^−1^. The adsorption of W(VI) ions onto PVC-TEAC anionite was found to be quite efficient, with R_L_ values between 0.0041 and 0.00052. For the adsorption, the Freundlich isotherm model was also used [60,61]. While fundamentally mathematical, this equation is frequently helpful when trying to make sense of facts. The Freundlich isotherm model is represented by the following equation:


(8) $$Log\;q_{e}=Log\;K_{f}+\left(\frac{1}{n}\right).$$

In this equation, C_e_ is the equilibrium concentration (mg L^−1^) and q_e_ is the sum of W(VI) ions adsorbed at equilibrium, K_f_ is the adsorption uptake capacity in mg/g, and n is the adsorption rate. Based on a linear relationship between Log q_e_ and Log C_e_, the values of K_f_ and n were determined to be 52.335 mg g^−1^ and 3, respectively. Adsorption is preferable between the numbers 0 and 10. According to Figure 12c, the K_f_ value (52.335 mg g^−1^) is less than the experimental result, and the Freundlich plot correlation coefficient is R^2^ = 0.9084. This suggests that Langmuir’s theory is a better fit for the data than Freundlich. The parameters of the isotherm for W(VI) adsorption onto PVC-TEAC anionite are shown in Table 5.

#### 3.3.4. The effect of PVC-TEAC anionite dose

The impact of PVC-TEAC anionite dosage on the uptake of W(VI) ions has been investigated, and the results are presented in Figure 12d. The following observations can be made: (1) Increase in dosage (0.01 g to 0.05 g); As the dosage of PVC-TEAC anionite increases from 0.01 g to 0.05 g, there is a corresponding increase in the absorption of W(VI) ions. This suggests that higher amounts of PVC-TEAC anionite result in a higher uptake of tungsten ions. (2) Decrease in dosage (0.05 g to 0.5 g): However, as the dosage is further increased from 0.05 g to 0.5 g, the absorption of W(VI) ions progressively declines. This decline occurs because the number of active sites on the PVC-TEAC anionite becomes excessive, surpassing the total amount of W(VI) ions available for adsorption. (3) Breakthrough point: The findings indicate that 0.05 g of PVC-TEAC anionite represents a critical point. At this dosage, the total uptake capacity reaches 126 mg g^−1^, which is equivalent to 126 mg L^−1^ of W(VI) ions. This suggests that 0.05 g of PVC-TEAC anionite is an optimal dosage for achieving maximum absorption efficiency under the specified conditions. Understanding the dosage effect is crucial for practical applications, as it helps determine the appropriate amount of PVC-TEAC anionite needed for efficient W(VI) ion removal while avoiding excess usage beyond the saturation point.

#### 3.3.5. Thermodynamic prospects

**Figure 13:**
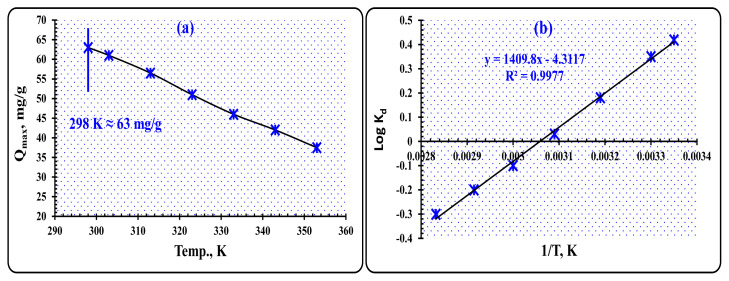
(a) The effect of temperature on W(VI) ions uptake by PVC-TEAC anionite (b) The effect of temperature on the partition coefficient of W(VI) ions using PVC-TEAC anionite. *(Conditions: V: 25 mL, W(VI) conc.: 150 mg L*^−^*^1^, m: 0.05 g, pH 8, agitation time : 15 min).*

The effect of temperature on the adsorption equilibrium and spontaneity of the adsorption process at different temperatures can be described using the significant thermodynamic parameters obtained from both Vant-Hoff and Gibbs free energy calculations. To determine the impact of temperature on the uptake of W(VI) ions, a solution containing 0.05 g of PVC-TEAC anionite and 25 mL of aqueous tungsten solution with a concentration of 150 mg L^−1^ at pH 8 was used. The contact time was set at 15 min, and the temperatures ranged from 298 to 353 K. It was observed that as the temperature increased from 298 to 353 K, the uptake capacity decreased from 63 to 37.5 mg g^−1^, respectively. This influence of temperature on W(VI) ions uptake is depicted in Figure 13a, indicating that the adsorption process of W(VI) ions onto PVC-TEAC anionite is an exothermic process.

In order to determine the thermodynamic parameters, such as Gibbs free energy (ΔG, kJ mol^−1^), enthalpy change (ΔH, kJ mol^−1^), and entropy change (ΔS, J mol^−1^ K^−1^), specific formulae were employed. These formulae allowed for accurate measurements and calculations of these crucial parameters. By utilizing these calculations, scientists and researchers were able to gain valuable insights into the energy changes and transformations that occur in chemical reactions and processes [62]:


(9) $$\Delta G=-2.303\;RT\;Log\;K_{d},$$

(10) $$Log\;K_{d}=\frac{\Delta \;S}{2.303\;R}-\frac{\Delta H}{2.303\;RT}.$$

The universal gas constant, represented by R (8.314 J mol^−1^ K^−1^), and the temperature in Kelvin (K), represented by T, are used to calculate the values of ΔH and ΔS. Figure 13b demonstrates that these values can be determined mathematically by analyzing the slope and intersection of the Log K_d_ against 1/T plot. The slope is calculated to be 1409.8, while the intersection is −4.3117. The correlation coefficient, R^2^, is found to be 0.9977. This information is crucial in understanding the thermodynamics of the system being studied and can aid in predicting future behavior. It is important to note that accurate calculations of these values are essential for making informed decisions regarding the system in question.

The results presented in Table 6 indicate that the preservation of W(VI) ions onto PVC-TEAC anionite is an exothermic process, as evidenced by the negative ΔH value of −27 kJ mol^−1^. The adsorption process also shows a slight decrease in randomness, as indicated by the negative ΔS value of −0.082 kJ mol^−1^. The negative ΔG values observed at temperatures between 298 and 323 K suggest that the adsorption mechanism is thermodynamically spontaneous and feasible at low temperatures. Furthermore, the increase in ΔG values with increasing temperature indicates that the adsorption is more favorable at lower temperatures. The Arrhenius equation is a valuable tool for determining the apparent activation energy (E_a_) of W(VI) ion adsorption onto PVC-TEAC anionite at different temperatures. By calculating the slope of the straight line produced in Figure 13b, the Arrhenius equation can be used to estimate E_a_ [63]:


(11) $$Log\;K_{d}=\frac{-2.303\;E_{a}}{RT}+Log\;A.$$

The partition coefficient, K_d_, the adsorption activation energy, E_a_ (kJ mol^−1^), the molar gas constant, R (8.314 J mol^−1^ K^−1^), temperature, T (in Kelvin), and the preexponential factor, A, which is independent of temperature, are all significant factors in understanding the adsorption of W(VI) ions. By calculating the activation energy required for W(VI) ion adsorption as −5.09 kJ mol^−1^, it is evident that the process of adsorption onto PVC-TEAC anionite is exothermic and occurs spontaneously at room temperature. In this case, there is no need for additional activation energy, and the adsorption process is not highly influenced by temperature variations. These findings shed light on the nature of the adsorption process and provide valuable insights for further research in this field.

#### 3.3.6. The separation factor parameter (S.F.)

The investigation focused on identifying the potential foreign ions present in the leach liquor when studying coions with tungsten. It is important to note that the alkali fusion method employed for opening tungsten minerals effectively eliminated most foreign ions as insoluble hydroxides, leaving behind only soluble sodium tungstate (Na_2_WO_4_) in the leach liquor. Consequently, the concentration of foreign ions in the leach liquor is expected to be relatively low. To assess the impact of individual foreign ions, a series of experiments were conducted under optimal conditions. Each foreign ion was introduced individually into a 25-mL aqueous boron solution with a concentration of 150 mg L^−1^, at a pH of 8. The solution was then agitated with 0.05 g of PVC-TEAC anionite at 25 °C for 15 min. The efficiency and selectivity of PVC-TEAC anionite towards tungstate ions were evaluated using the separation factor parameter (S.F*). The results, as shown in Table 7, indicated that under the specified optimum conditions, PVC-TEAC anionite demonstrated satisfactory adsorption of tungstate ions with a favorable separation factor compared to other foreign ions. Notably, metal cations such as Na^+^, K^+^, Ca^2+^, Ni^2+^, Pb^2+^, Zn^2+^, and Mg^2+^ exhibited high separation factors. Conversely, heavy metals including Fe^3+^, Cr^3+^, Mn^2+^, V^5+^, Si^4+^, and Al^3+^ had somewhat adverse effect on the adsorption of tungstate ions, resulting in different separation factor values. It is worth mentioning that PVC-TEAC anionite exhibits a strong affinity for anionic complex species formed in the leach liquor, while cationic complex species do not display the same affinity.

#### 3.3.7. Tungsten elution

The elution of W(VI) from the loaded PVC-TEAC anionite was investigated using three different mineral acids at various concentrations. The eluting agents, which ranged from 0.025 to 2 M, were tested at room temperature with a fixed acid volume of 10 mL for every 0.05 g of loaded PVC-TEAC anionite. The results, as presented in Table 8, revealed that the elution efficiency of W(VI) decreased at lower acidic concentrations, while it improved with higher acid concentrations. Remarkably, an elution efficiency of 99% was achieved using 1 M HNO_3_, 1 M HCl, and 2 M H_2_SO_4_ on the PVC-TEAC anionite. From an economic standpoint, sulfuric acid with a concentration of 1 M could be employed. This finding demonstrates the potential for efficient elution of W(VI) using mineral acids, particularly sulfuric acid, which offers a cost-effective solution.

#### 3.3.8. Tungsten precipitation

In order to obtain the appropriate tungsten concentrate (yellow tungsten oxide, WO_3_) from the eluted solution, certain steps must be taken. Firstly, the solution is adjusted to the desired pH using a 30% NH_4_OH and concentrated H_2_SO_4_ solution until it reaches a pH of 1. This is followed by boiling the solution on a hotplate for 3 min, after which it is allowed to cool. During this process, tungsten crystallizes and forms a yellow tungstic acid precipitate (WO_3_.H_2_O↓). The resulting precipitate is then separated through filtration and subjected to calcination in an electrical oven at a temperature of 600 °C. This final step yields the desired tungsten concentrate (WO_3_). To ensure the quality and characteristics of the tungsten oxide, various tests are conducted. These include XRD analysis to determine the crystal structure, FTIR analysis to identify functional groups, and ICP-OES and SEM-EDX analyses to assess chemical composition and surface morphology.

#### 3.3.9. Mineralogical and chemical composition of wolframite ore sample

One of the most significant and promising areas in Egypt is Gabal Qash Amir, situated in the extreme Southeastern part of the country. It is located approximately 28 km Southwest of Abu-Ramad city and is in close proximity to the Sudan border. The area is bounded by longitudes 36° 10′ 59″–36° 14′ 24″ E and latitudes 22° 14′ 07″–22° 15′ 21″ N. Gabal Qash Amir falls within the Arabian-Nubian shield zone, known for its abundance of valuable metals such as Mn, Zr, Ta, U, Nb, and W [64]. A sample of mineralized invading quartz vein was carefully collected from this area’s wolframite granite. To reduce the size of mineral particles to less than 1 mm, a bulk sample weighing 10 kg (containing around 0.43% w/w of WO_3_) was ground using a roll mill crusher and jaw crushers. The gravity concentration process involved utilizing a lab wet shaking table, which resulted in the production of a tungsten-rich concentrate. To further enhance preconcentration, a magnetic separation technique was employed with the aid of a high-intensity induced magnetic roll separator to recover wolframite-rich minerals.

**Figure 14:**
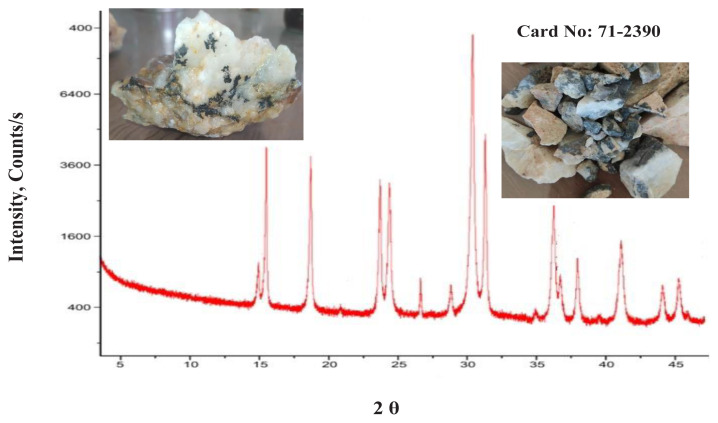
XRD diffractogram of wolframite ore sample.

**Figure 15:**
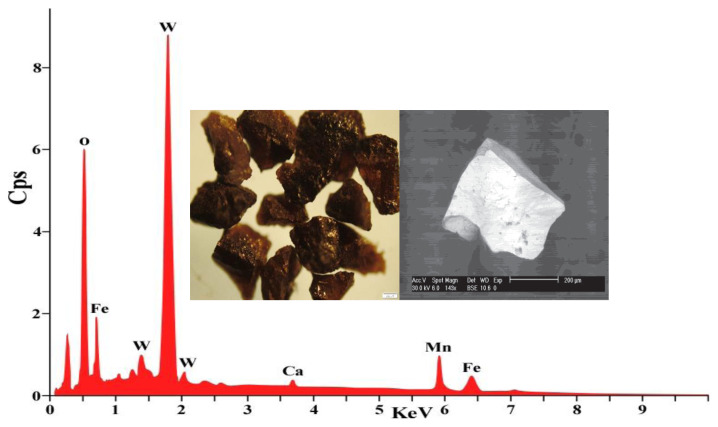
SEM-EDX analysis of wolframite ore sample.

Wolframite, a mineral composed mainly of manganese tungsten oxide (Fe,Mn)WO_4_, serves as the transitional mineral between ferberite (rich in iron) and hübnerite (rich in manganese). The semiquantitative analysis of a wolframite sample reveals a significant presence of tungsten and manganese elements, accounting for 88.8% of the sample’s weight. Additionally, the sample contains noteworthy amounts of silicate and iron. To further examine the Wolframite sample, various analytical techniques were employed, including X-ray diffraction (XRD) (Figure 14), scanning electron microscopy with energy-dispersive X-ray spectroscopy (SEM-EDX) (Figure 15), and inductively coupled plasma optical emission spectroscopy (ICP-OES) (Table 9).

The wolframite ore sample underwent a thorough chemical analysis subsequent to the physical separation process, also known as the preconcentration step. This analysis revealed the presence of several key components in the sample. The primary component identified was WO_3_, which accounted for 70.91% of the sample. Additionally, the sample contained 6.55% MnO and 21.1% FeO. On the other hand, minor trace elements such as SiO_2_, Al_2_O_3_, CaO, and MgO were also detected in the sample, as indicated in Table 9. These findings provide a comprehensive understanding of the chemical composition of the Wolframite ore sample, enabling further insights into its potential applications and properties.

#### 3.3.10. Dissolution of Wolframite ore sample

The technique of alkali fusion leaching, employing sodium hydroxide flux, is utilized to release tungsten from the Gabal Qash Amir Wolframite ore sample found in the Southeastern Desert of Egypt. This process involves converting the ore into soluble sodium tungstate through fusion with sodium hydroxide, followed by leaching with hot water. Initially, the wolframite ore is ground and sieved to a grain size of 74 μm (–200 mesh). The fusion process is then optimized by subjecting the mixture to a 3-h fusion time in an electrical oven at a temperature of 850 °C, using a mass ratio of alkali to ore of 2/1. After cooling, the resulting fused product is ground and sieved to the same grain size and subsequently subjected to hot water leaching for 30 min, with a solid to liquid phase ratio of 1g/100 mL. The efficiency of tungsten leaching achieved through alkali fusion technology is an impressive 99%, thanks to the optimization of various leaching factors [20,65].

Following the hot water leaching process, it is crucial to eliminate any remaining metal hydroxides precipitation through solid/liquid separation, as these by-products can interfere with the prepared alkaline leach liquor. Alkali fusion technique, coupled with water leaching, has been shown to produce leach liquor that is rich in tungsten (sodium tungstate), while other impurities precipitate as insoluble hydroxides. To further refine the product, a physical separation step using gravity concentration was employed, which involved the use of a lab wet shaking table. This operation allowed for the production of a tungsten-rich concentrate. Preconcentration was also achieved through magnetic separation, utilizing a high-intensity induced magnetic roll separator to recover Wolframite-rich mineral with very low impurities such as SiO_2_ and Al_2_O_3_. After solid/liquid separation, the raffinate containing tungsten could be extracted onto PVC-TEAC anionite. Overall, these techniques are essential in ensuring the production of high-quality tungsten products.

#### 3.3.11. Application: tungsten recovery from Qash Amir, wolframite ore sample, Southeastern Desert of Egypt, by PVC-TEAC anionite

The optimized data obtained from the experiment allowed for the successful extraction of tungsten from a Wolframite ore sample using PVC-TEAC anionite. The experiment was conducted under specific conditions, including a pH of 8, agitation time of 15 min, and a temperature of 25 °C. The extraction process involved the agitation of 1 L of the leach liquor with 25 g of PVC-TEAC anionite until the maximum uptake capacity of 63 mg g^−1^ was reached. Results showed that the anionite had a tungsten extraction efficiency of 99%, and the extracted tungsten was easily eluted by 250 mL of 1M H_2_SO_4_ within 10 min to obtain tungstic acid. Further purification was achieved through redissolving the product in distilled H_2_O and directing it back to PVC-TEAC anionite. The eluate rich tungsten solution was then recrystallized, boiled, and evaporated to produce high-pure tungstic acid (WO_3_.H_2_O↓), which was calcinated in an electrical oven for 3 h at 600 °C to obtain tungsten oxide concentrate as the final product.

**Figure 16:**
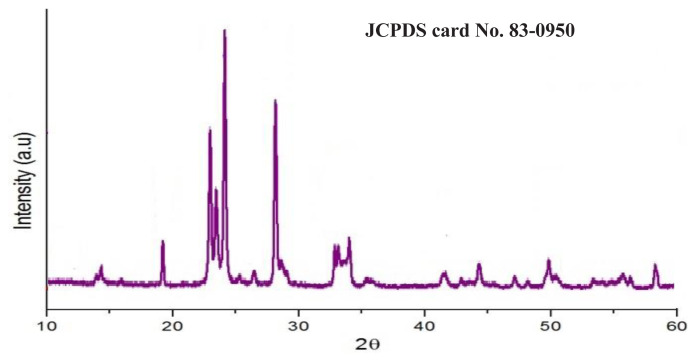
XRD diffractogram of pure WO_3_ concentrate produced by PVC-TEAC anionite.

**Figure 17:**
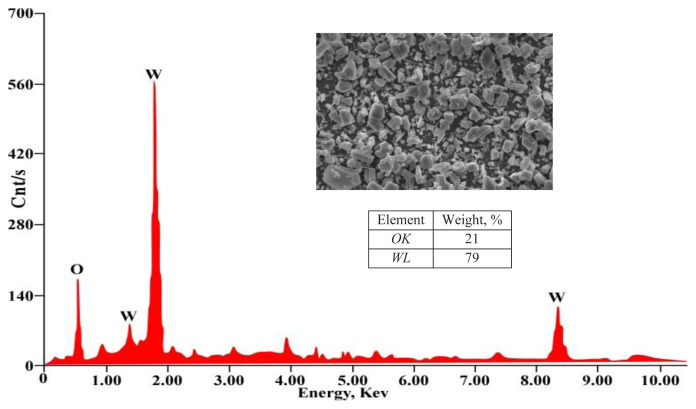
SEM-EDX of pure WO_3_ concentrate produced by PVC-TEAC anionite.

**Figure 18:**
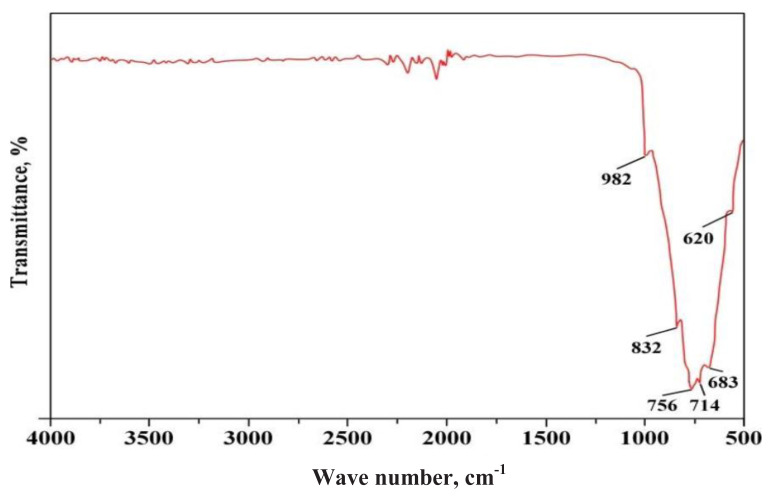
FT-IR spectra of pure WO_3_ concentrate produced by PVC-TEAC anionite.

The tungsten content and the presence of metal ion impurities in tungstic acid concentrate are evaluated through various analytical techniques, including XRD, SEM-EDX, FT-IR, and ICP-OES analysis. The findings of these analyses are presented in Figures 16–18 and Table 10, respectively. Based on the results obtained, it can be concluded that the tungsten content in the tungsten oxide concentrate produced by PVC-TEAC anionite is 78.3%, achieving a purity level of 98.75%.

The X-ray diffraction (XRD) analysis of the tungsten oxide crystal is illustrated in Figure 16. The crystal structure of the tungsten oxide concentrate corresponds to the monoclinic phase of WO_3_, as confirmed by JCPDS card No. 83-0950. The diffraction pattern reveals distinct and intense peaks at specific 2θ angles, namely 19.1°, 22.9°, 23.4°, 24.1°, 26.4°, 28.1°, 32.8°, 33.1°, 33.9°, 41.6°, 49.82°, and 50.43°. These peaks can be attributed to the (011), (002), (020), (200), (120), (112), (022), (202), (220), (222), (232), and (114) crystalline planes of the monoclinic phase of WO_3_, indicating a well-defined crystalline structure. Notably, the diffraction peaks of tungsten oxide are sharp and devoid of any impurity peak, signifying a high level of purity and an orderly crystal lattice for WO_3_ [66]. Furthermore, the SEM-EDX analysis shown in Figure 17 assures the high purity of WO_3_ concentrate as only W and O elements are found assaying 79% and 21% for W and O elements, respectively.

To determine the composition and crystal structure of the sample, an FT-IR measurement was conducted. The infrared spectrum of monoclinic WO_3_ exhibited a significant band with characteristic frequency vibrations ranging from 400 to 1000 cm^−1^ (Figure 18). Within this range, the band at 982 cm^−1^ was identified as metal-oxygen vibrations, specifically related to the stretching vibrations of the W=O bond. Another band at 620 cm^−1^ was attributed to W-O stretching vibrations, while the band at 714 cm^−1^ was assigned to W-O-W bridging modes of the WO_6_ (octahedral) corner-sharing species. Notably, two additional bands at 832 and 756 cm^−1^ were observed, corresponding to interbridge stretching O-W-O and corner-sharing mode W-O-W, respectively. Moreover, the absence of a broad band around 3380 cm^−1^, which signifies H_2_O stretching vibration, indicated that the annealed WO_3_ sample did not retain any adsorbed water. This FT-IR spectrum provided valuable insights into the sample’s composition and confirmed the absence of water [67].

A comparative study for different composites for tungsten extraction from different matrices is shown in Table 11.

**Figure 19:**
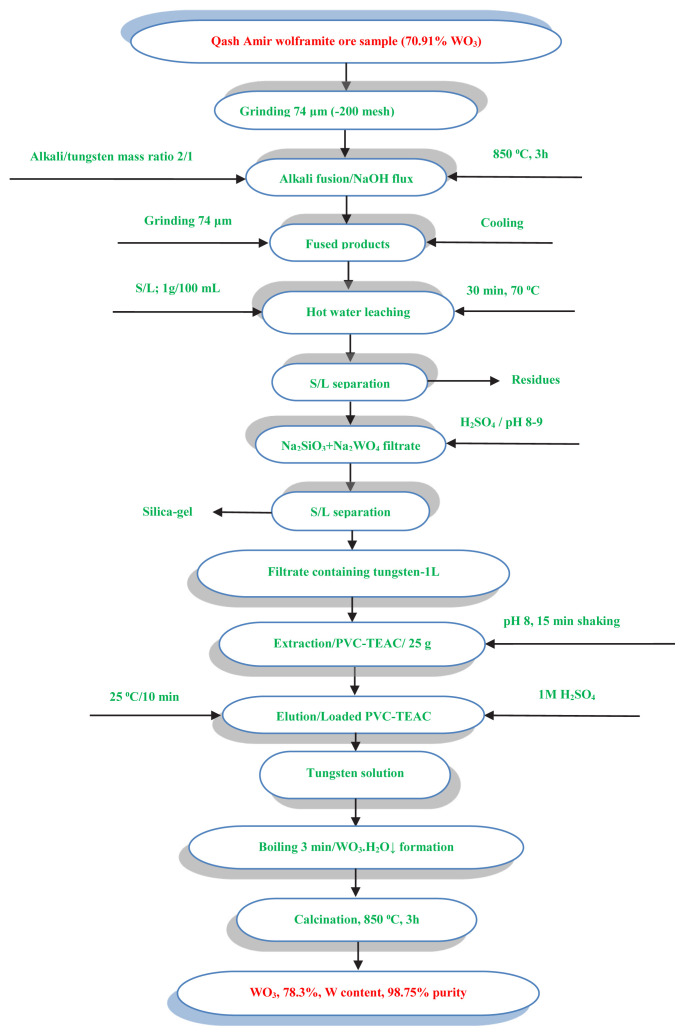
A flow chart with tungsten mass balance for the preparation of pure WO_3_ from Gabal Qash Amir, Southeastern Desert, Egypt, using PVC-TEAC anionite.

To illustrate the recovery process of tungsten from the Qash Amir wolframite ore sample in the Southeastern Desert of Egypt, Figure 19 depicts a schematic flow chart. This flow chart provides a visual representation of the steps involved in extracting tungsten from the ore sample. These comprehensive analyses and visual representations contribute to a better understanding of the tungsten content and purification process, facilitating further research and development in the field.

## 4. Conclusion

A highly efficient method for extracting tungsten ions from Gabal Qash Amir has been developed using a synthesized polyvinyl chloride functionalized triethanol ammonium chloride anionite (PVC-TEAC). Gabal Qash Amir is located approximately 28 km Southwest of Abu-Ramad city, near the Sudan border, with coordinates ranging from longitudes 36d 10′ 59″–36° 14′ 24″ E and latitudes 22° 14′ 07″–22° 15′ 21″ N. The tungsten content in the ore was determined to be 70.91% WO_3_ after physical preconcentration. Various characterization techniques such as FT-IR, XPS, BET, EDX, TGA, ^1^H-NMR, ^13^C-NMR, GC-MS, XRD, and ICP-OES were successfully employed to analyze the synthesized PVC-TEAC anionite. By optimizing the static adsorption technique with a 25-mL solution containing 150 mg L^−1^ of tungsten ions, agitated with 0.05 g of PVC-TEAC anionite at pH 8 for 15 min at ambient temperature, a maximum uptake of 63 mg g^−1^ was achieved at 25 °C. This was deemed economically viable. Kinetic modeling data demonstrated that both first- and second-order models accurately described the adsorption system, with theoretical retention capacities of 63.6 and 64.1 mg g^−1^, respectively, closely matching the realistic value of 63 mg g^−1^. The positive value of I (thickness of the boundary layer) indicated that the extraction of tungsten ions by PVC-TEAC anionite was regulated by an intraparticle diffusion mechanism.

The Langmuir isotherm model is the preferred method for understanding distribution isotherm modelling, as it provides the closest uptake value to practical applications at 61.728 mg g^−1^. Thermodynamic profiles indicate that the adsorption process is exothermic, spontaneous, and advantageous at low temperatures, with consideration given to ΔS (–0.082 kJ mol^−1^), ΔH (–27 kJ mol^−1^), and ΔG. As temperature increases, ΔG values also rise from −2.39 kJ mol^−1^ at 298 K to 2.034 kJ mol^−1^ at 353 K. Tungsten ions can be efficiently eluted from the loaded PVC-TEAC using 1M H_2_SO_4_ at a 97% efficiency rate. The PVC-TEAC anionite demonstrates a good separation factor (S.F.) towards most coions. A successful Alkali fusion with NaOH flux followed by extraction with PVC-TEAC anionite yields a high purity tungsten oxide concentrate (WO_3_) with a tungsten content of 78.3% and purity of 98.75%.

## Supplementary Data

Figure S1 BET analysis of (a, b) PVC, (c, d) PVC-TEAC (e, f),PVC-TEAC-W.

## Figures and Tables

**Table 1:** Chemical composition of PVC, PVC-TEAC, and PVC-TEAC-W.

Sample	C*_1S_*, %	O*_1S_*, %	N*_1S_*, %	Cl*_2P_*, %	W*_4F_*, %
PVC	38.02	−	-	56.37	-
PVC-TEAC	47.57	19.5	17.31	15.6	-
PVC-TEAC-W	46.5	19.45	17.3	15.5	1.25

**Table 2:** The surface area (S _BET_), specific pore volume (V), and the average pore diameter (d) of PVC, PVC-TEAC, and PVC-TEAC-W.

Sample	S _BET_, m^2^ g^−1^	V, cm^3^ g^−1^	d, nm
PVC	24.75	0.0262	1.97
PVC-TEAC	45.14	0.0935	2.1
PVC-TEAC-W	43.068	0.0889	2

**Table 3:** TGA properties of PVC, PVC-TEAC, and PVC-TEAC-W composites.

Samples	PVC		PVC-TEAC		PVC-TEAC-W	
TGA stages	Temp., °C	Weight loss, %	Temp., °C	Weight loss, %	Temp., °C	Weight loss, %
1st	0–100	5	0–100	5	0–100	5
2nd	100–250	13	100–265	19	100–345	15
3rd	250–450	64	265–500	40	345–500	36
4th	450–600	12.5	500–600	30	500–600	33
Final residue	600–800	5.5	600–800	8	600–800	13

**Table 4:** Kinetic parameters of W(VI) adsorption upon PVC-TEAC anionite.

Experimental capacity q_max_, mg g^−1^	Pseudofirst-order			Pseudosecond-order			Intraparticle diffusion		
63 mg g^−1^	q_e_	K_1_	R^2^	q_e_	K_2_	R^2^	K_ad_	I	R^2^
	63.6	0.428	0.9965	64.1	0.0238	0.9994	10.813	26.805	0.7768

**Table 5:** Isotherm parameters of W(VI) adsorption upon PVC-TEAC anionite.

Experimental capacity q_max_, mg g^−1^	Langmuir model			Freundlich model		
63 mg g^−1^	q_e_	b	R^2^	K_f_	n	R^2^
	61.728	9.523	0.999	52.335	3	0.9084

**Table 6:** The thermodynamic indices of W(VI) ions adsorption upon PVC-TEAC anionite.

Parameter	ΔH , kJ mol^−1^	ΔS, kJ mol^−1^ K^−1^		ΔG, kJ mol^−1^					
W(VI)	−27	−0.082	298 K	303 K	313 K	323 K	333 K	343 K	353 K
			−2.39	−2.03	−1.078	−0.185	0.637	1.313	2.034

**Table 7:** The effect of foreign ions on tungsten adsorption using PVC-TEAC anionite.

Co-ions	Feed solution, mg L^−1^	Raffinate solution, mg L^−1^	S.F*	Coions	Feed solution, mg L^−1^	Raffinate solution, mg L^−1^	S.F*
W^6+^	150	24	-	Fe^3+^	1000	750	378.37
Na^+^	1000	1000	175×10^3^	Ti^4+^	1000	950	2.4×10^3^
K^+^	1000	1000	175×10^3^	Ca^2+^	1000	1000	175×10^3^
Si^4+^	1000	700	294.4	Mg^2+^	1000	1000	175×10^3^
Al^3+^	1000	750	378.37	V^5+^	1000	650	234.2
Mn^2+^	1000	800	504	Cr^3+^	1000	700	294.4
Fe^2+^	1000	1000	175×10^3^	Zn^2+^	1000	980	6.3×10^3^
Pb^2+^	1000	950	3.325×10^3^	Ni^2+^	1000	980	6.3×10^3^

**(Adsorption conditions:** pH: 8, V: 25 mL, m; 0.05 g, Agitation time: 15 min., W (VI): 150 mg L^−1^, temp. : 25 °C)

* **Separation factor (S.F):** the distribution coefficient of tungsten ions (D_W_) over partition coefficient of foreign ions (D_M_).

**Table 8:** Effect of eluting agents concentration on W(VI) elution from loaded PVC-TEAC anionite.

Acid concentration, M	Elution efficiency, (%)		
	HNO_3_	HCl	H_2_SO_4_
0.025	62.5	54.5	57
0.05	84.5	66	63
0.1	90	76	71
0.5	95	90	81
1	99	99	97
2	99	99	99

**Table 9:** Chemical composition of Gabal Qash Amir Wolframite ore sample by ICP-OES.

Component	Content , %
WO_3_	70.91
MnO	6.55
FeO	21.1
CaO	0.28
MgO	0.45
SiO_2_	0.13
Al_2_O_3_	0.34

**Table 10:** ICP-OES analysis of pure tungsten oxide concentrate.

Element	Content, %	Element	Content, %
W	78.3	Mg	0.0018
Si	0.15	Ti	0.028
Al	0.066	V	0.151
Na	0.0017	Cr	0.26
K	0.0015	Mn	0.311
Ca	0.0022	Zn	0.0059

**Table 11:** Different composites for tungsten recovery from different matrices.

Composite	Q_max_, mg g^−1^	Ref.
Fe-HT3	54.6	[68]
Fe-HT5	49	[68]
Al-HT3	22	[68]
Al-HT5	35.7	[68]
MgAl-LDH (Layered double hydroxide)	61.28	[35]
Anionite AV-17-8	35.23	[40]
D403	11.9	[32]
TEVA	18.8	[69]
Lewatit Monoplus MP600	78.5	[39]
PVC-TEAC	63	Present study
