# Supplementary figures and images for: Synthesis, characterization, and physico-chemical aspects of a new PVC-based quaternary triethanol ammonium chloride anionite for tungsten recovery
Source: Turk J Chem. 2024 Mar 13;48(4):524–49. doi: 10.55730/1300-0527.3678 (PMC11407338; doi:10.55730/1300-0527.3678)

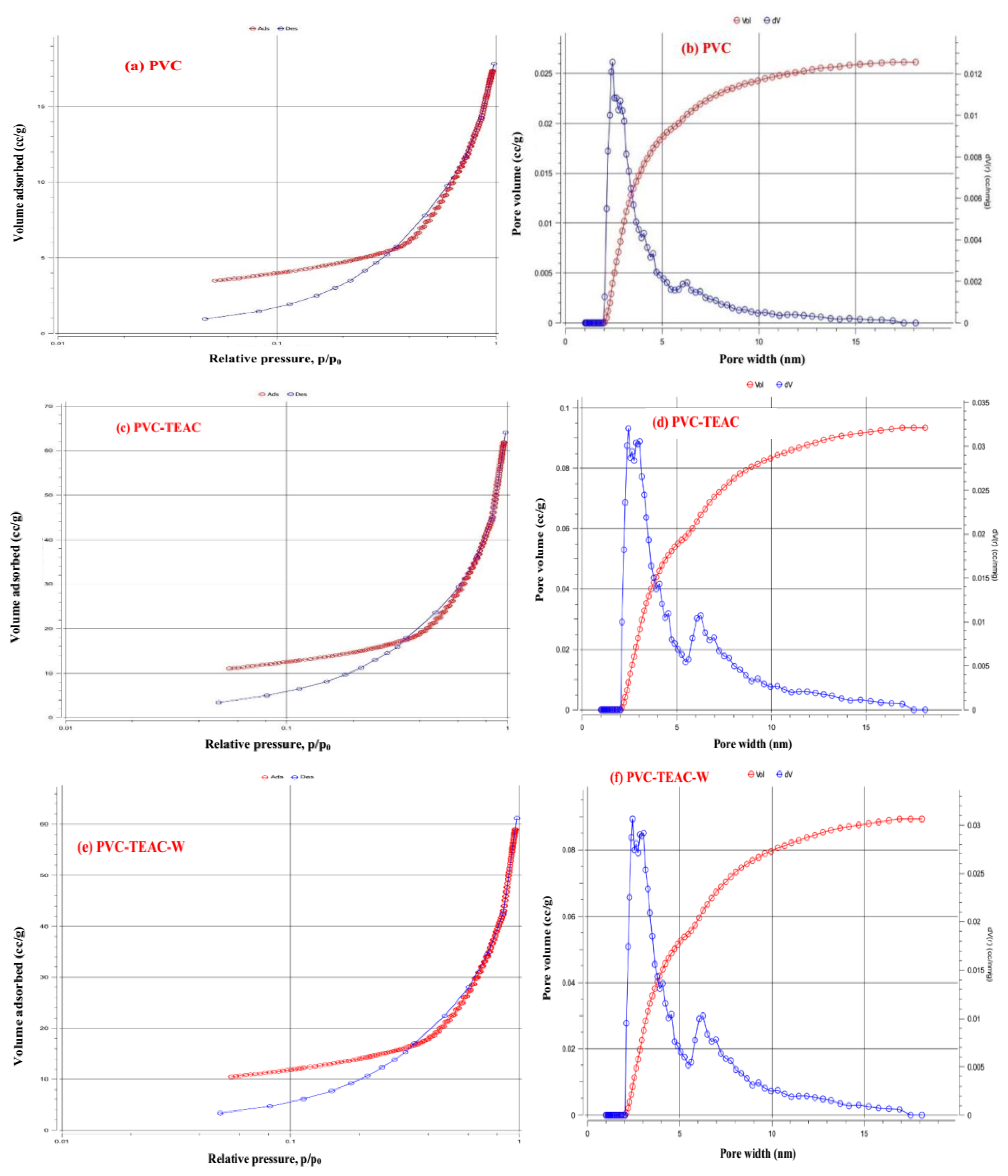

Supplement: Figure S1 — BET analysis of (a, b) PVC, (c, d) PVC-TEAC (e, f),PVC-TEAC-W. [file tjc-48-04-524s1.tif]
